# Optimal Determination of Respiratory Airflow Patterns Using a Nonlinear Multicompartment Model for a Lung Mechanics System

**DOI:** 10.1155/2012/165946

**Published:** 2012-06-08

**Authors:** Hancao Li, Wassim M. Haddad

**Affiliations:** School of Aerospace Engineering, Georgia Institute of Technology, Atlanta, GA 30332-0150, USA

## Abstract

We develop optimal respiratory airflow patterns using a nonlinear multicompartment model for a lung mechanics system. Specifically, we use classical calculus of variations minimization techniques to derive an optimal airflow pattern for inspiratory and expiratory breathing cycles. The physiological interpretation of the optimality criteria used involves the minimization of work of breathing and lung volume acceleration for the inspiratory phase, and the minimization of the elastic potential energy and rapid airflow rate changes for the expiratory phase. Finally, we numerically integrate the resulting nonlinear two-point boundary value problems to determine the optimal airflow patterns over the inspiratory and expiratory breathing cycles.

## 1. Introduction

Respiratory failure, the inadequate exchange of carbon dioxide and oxygen by the lungs, is a common clinical problem in critical care medicine, and patients with respiratory failure frequently require support with mechanical ventilation while the underlying cause is identified and treated. The goal of mechanical ventilation is to ensure adequate ventilation, which involves a magnitude of gas exchange that leads to the desired blood level of carbon dioxide, and adequate oxygenation, which involves a blood concentration of oxygen that will ensure organ function. Achieving these goals is complicated by the fact that mechanical ventilation can actually cause acute lung injury, either by inflating the lungs to excessive volumes or by using excessive pressures to inflate the lungs. The challenge to mechanical ventilation is to produce the desired blood levels of carbon dioxide and oxygen without causing further acute lung injury.

With the increasing availability of microchip technology, it has been possible to design partially automated mechanical ventilators with control algorithms for providing volume or pressure control [1–5]. More sophisticated fully automated model reference adaptive control algorithms for mechanical ventilation have also been recently developed [6, 7]. These algorithms require a reference model for identifying a clinically plausible breathing pattern. However, the respiratory lung models that have been presented in the medical and scientific literature have typically assumed homogenous lung function. For example, in analogy to a simple electrical circuit, the most common model has assumed that the lungs can be viewed as a single-compartment characterized by its compliance (the ratio of compartment volume to pressure) and the resistance to air flow into the compartment [8–10]. While a few investigators have considered two compartment models, reflecting the fact that there are two lungs (right and left), there has been little interest in more detailed models [11–13].

Early work on the optimality of respiratory control mechanisms using simple homogenous lung models dealt with the frequency of breathing. In particular, the authors in [14–17] predicted the frequency of breathing by using a minimum work-rate criterion. This work involves a static optimization problem and assumes that the airflow pattern is a fixed sinusoidal function. The authors in [17, 18] developed optimality criteria for the prediction of the respiratory airflow pattern with fixed inspiratory and expiratory phases of a breathing cycle. These results were extended in [19] by considering a two-level hierarchical model for the control of breathing, in which the higher-level criterion determines values for the overall control variables of the optimal airflow pattern derived from the lower-level criteria, and the lower-level criteria determine the airflow pattern with the respiratory parameters chosen by minimizing the higher-level criterion.

Although the problem for identifying optimal respiratory patterns has been addressed in the literature (see [14, 16–19] and the references therein), the models on which these respiratory control mechanisms have been identified are predicated on a single compartment lung model with constant respiratory parameters. However, the lungs, especially diseased lungs, are heterogeneous, both functionally and anatomically, and are comprised of many subunits, or compartments, that differ in their capacities for gas exchange. Realistic models should take this heterogeneity into account. In addition, the resistance to gas flow and the compliance of the lung units are not constant but rather vary with lung volume. This is particularly true for compliance. While more sophisticated models entail greater complexity, since the models are readily presented in the context of dynamical systems theory, sophisticated mathematical tools can be applied to their analysis. Compartmental lung models are described by a state vector, whose components are the volumes of the individual compartments.

A key question that arises in the consideration of nonlinear multicompartment models is whether or not experimental data support a complex model. This question can be addressed by considering an analogy to pharmacokinetics. Specifically, the earliest pharmacokinetic models were typically one-compartment models. This reflected the challenges of sampling and drug assay. These models were adequate for quantifying drug disposition on a long time scale. For example, simple one-compartment models were adequate in describing the total clearance or volume of distribution. However, for even open-loop control of drug concentrations, the one compartment model was inadequate. More complex models (two- and three-compartment models) were needed that accounted for distribution as well as elimination processes (see [20] and the references therein).

 Similarly, for adaptive control of mechanical ventilation, that is, more advanced controller architectures than simple volume- or pressure-controlled ventilation, more elaborate models are needed, especially when accounting for nonlinear compliance and resistance and lung heterogeneity [6]. In the case of pharmacokinetics, the control algorithm can only be as complex as the data supports. This is also true for control of mechanical ventilation. Flow and pressure patterns in the airway are not simple waveforms, although clinicians to date have modeled them as such. There is considerable information embedded in these waveforms. The purpose of our work in this paper is to provide a mathematically rigorous and general framework developing optimal determination of respiratory airflow patterns using a nonlinear multicompartment model for a lung mechanics system. It is a an easy task to simplify this framework to be congruent with the granularity of the data. The reverse process, however, is not possible without the development of a general framework.

In this paper, we extend the work of [17, 18] to develop optimal respiratory airflow patterns using a nonlinear multicompartment model for a lung mechanics system. (The usage of the word *optimal* throughout the paper refers to an optimal solution of the calculus of variations problems addressed in the paper and not an optimal breathing pattern in the sense of respiratory physiology.) First, we extend the linear multicompartment lung model given in [6] to address system model nonlinearities. Then, we extend the performance functionals developed in [17, 18] for the inspiratory and expiratory breathing cycles to derive an optimal airflow pattern using classical calculus of variations techniques. In particular, the physiological interpretation of the optimality criteria involves the minimization of work of breathing and lung volume acceleration for the inspiratory breathing phase, and the minimization of the elastic potential energy and rapid airflow rate changes for the expiratory breathing phase. Finally, we numerically integrate the resulting nonlinear two-point boundary value problems to determine the optimal airflow patterns over the inspiratory and expiratory breathing cycles.

The notation used in this paper is fairly standard. Specifically, ℝ^*n*^ denotes the set of *n* × 1 real column vectors, and ℝ^*n*×*m*^, denotes the set of *n* × *m* real matrices. For *x* ∈ ℝ^*n*^ we write *x* ≥ ≥0 (resp., *x* ≫ 0) to indicate that every component of *x* is nonnegative (resp., positive). In this case, we say that *x* is *nonnegative* or *positive*, respectively. Likewise, *A* ∈ ℝ^*n*×*m*^ is *nonnegative* or *positive* if every entry of *A* is nonnegative or positive. (In this paper, it is important to distinguish between a square nonnegative (resp., positive) matrix and a nonnegative-definite (resp., positive-definite) matrix.) Furthermore, ${\overset{̅}{\mathbb{R}}}_{+}^{n}$ and ℝ_+_^*n*^ denote the nonnegative and positive orthants of ℝ^*n*^, that is, if *x* ∈ ℝ^*n*^, then $x\in {\overset{̅}{\mathbb{R}}}_{+}^{n}$ and *x* ∈ ℝ_+_^*n*^ are equivalent, respectively, to *x* ≥ ≥0 and *x* ≫ 0. Finally, we write (·)^*T*^ to denote transpose, (·)′ to denote Frèchet derivative, and *δx* to denote the first variation of the function *x*.

## 2. A Nonlinear Multicompartment Modelfor Respiratory Dynamics

 In this section, we extend the linear multicompartment lung model of [6] to develop a nonlinear model for the dynamic behavior of a multicompartment respiratory system in response to an arbitrary applied inspiratory pressure. Here, we assume that the bronchial tree has a dichotomy architecture [21]; that is, in every generation each airway unit branches into two airway units of the subsequent generation. In addition, we assume that the lung compliance is a nonlinear function of lung volume.

**Figure 1:**
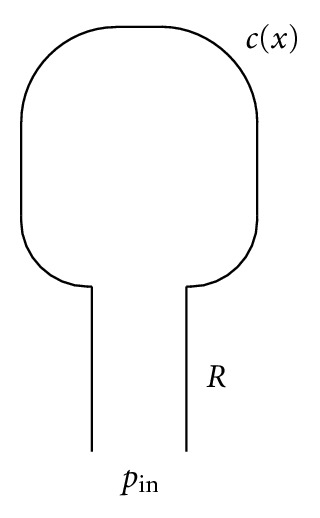
Single-compartment lung model.

First, for simplicity of exposition, we consider a single-compartment lung model as shown in Figure 1. In this model, the lungs are represented as a single lung unit with nonlinear compliance *c*(*x*) connected to a pressure source by an airway unit with resistance (to airflow) of *R*. At time *t* = 0, a driving pressure *p*_in_(*t*) is applied to the opening of the parent airway, where *p*_in_(*t*) is generated by the respiratory muscles or a mechanical ventilator. This pressure is applied over the time interval 0 ≤ *t* ≤ *T*_in_, which is the inspiratory part of the breathing cycle. At time *t* = *T*_in_, the applied airway pressure is released and expiration takes place passively, that is, the external pressure is only the atmospheric pressure *p*_ex_(*t*) during the time interval *T*_in_ ≤ *t* ≤ *T*_in_ + *T*_ex_, where *T*_ex_ is the duration of expiration.

The state equation for inspiration (inflation of lung) is given by

(1) $$\begin{array}{c} R_{\text{in}}\dot{x}\left(t\right)+\frac{1}{c_{\text{in}}\left(x\right)}x\left(t\right)=p_{\text{in}}\left(t\right),\;x\left(0\right)=x_{0}^{\text{in}},\;0\leq t\leq T_{\text{in}}, \end{array}$$

where *x*(*t*) ∈ ℝ, *t* ≥ 0, is the lung volume, *R*_in_ ∈ ℝ is the resistance to airflow during the inspiration period, *c*_in_ : ℝ → ℝ_+_ is a nonlinear function defining the lung compliance at inspiration, and *x*_0_^in^ ∈ ℝ_+_ is the lung volume at the start of the inspiration and serves as the system initial condition. Equation (1) is simply a pressure balance equation where the driving pressure *p*_in_(*t*),  0 ≤ *t* ≤ *T*_in_, applied to the compartment is proportional to the volume of the compartment via the compliance and the rate of change of the compartmental volume via the resistance. We assume that expiration is passive due to the elastic stretch of the lung unit. During the expiration process, the state equation is given by

(2) $$\begin{array}{c} R_{\text{ex}}\dot{x}\left(t\right)+\frac{1}{c_{\text{ex}}\left(x\right)\;}x\left(t\right)=p_{\text{ex}}\left(t\right),\;\;\;x\left(T_{\text{in}}\right)=x_{0}^{\text{ex}},\\ T_{\text{in}}\leq t\leq T_{\text{in}}+T_{\text{ex}}, \end{array}$$

where *x*(*t*) ∈ ℝ, *t* ≥ 0, is the lung volume, *R*_ex_ ∈ ℝ is the resistance to air flow during the expiration period, *c*_ex_ : ℝ → ℝ_+_ is a nonlinear function defining the lung compliance at expiration, and *x*_0_^ex^ ∈ ℝ_+_ is the lung volume at the start of expiration.

**Figure 2:**
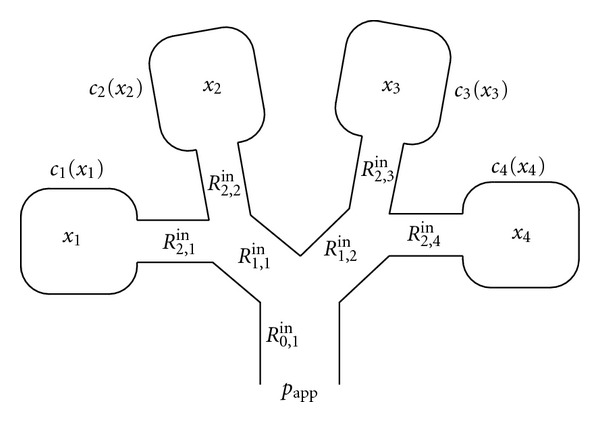
Four-compartment lung model.

Next, we develop the state equations for inspiration and expiration for a 2^*n*^-compartment model, where *n* ≥ 0. In this model, the lungs are represented as 2^*n*^ lung units which are connected to the pressure source by *n* generations of airway units, where each airway is divided into two airways of the subsequent generation leading to 2^*n*^ compartments (see Figure 2 for a four-compartment model).

Let *x*_*i*_, *i* = 1,2,…, 2^*n*^, denote the lung volume in the *i*th compartment, let *c*_*i*_^in^(*x*_*i*_) (resp., *c*_*i*_^ex^(*x*_*i*_)), *i* = 1,2,…, 2^*n*^, denote the compliance at inspiration (resp., expiration) of each compartment as a nonlinear function of the volume of *i*th compartment, and let *R*_*j*,*i*_^in^ (resp., *R*_*j*,*i*_^ex^), *i* = 1,2,…, 2^*j*^, *j* = 0,…, *n*, denote the resistance (to air flow) of the *i*th airway in the *j*th generation during the inspiration (resp., expiration) period with *R*_01_^in^ (resp., *R*_01_^ex^) denoting the inspiration (resp., expiration) of the *parent* (i.e., 0th generation) airway.

**Figure 3:**
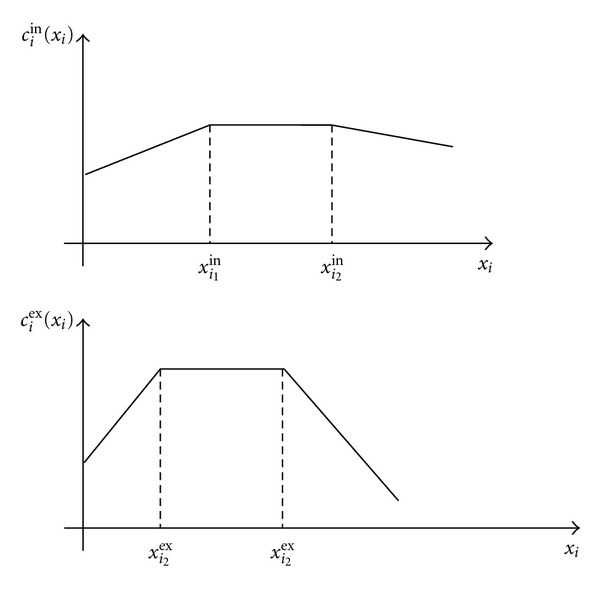
Typical inspiration and expiration compliance functions as function of compartmental volumes.

As in the single-compartment model, we assume that a pressure of *p*_in_(*t*), *t* ≥ 0, is generated (by the inspiratory muscles) or applied (by a mechanical ventilator) during inspiration. Now, the state equations for inspiration are given by

(3) $$\begin{array}{c} R_{n,i}^{\text{in}}{\dot{x}}_{i}\left(t\right)+\frac{1}{c_{i}^{\text{in}}\left(x_{i}\left(t\right)\right)}x_{i}\left(t\right)+\overset{n-1}{\underset{j=0}{\sum }}R_{j,k_{j}}^{\text{in}}\overset{k_{j}2^{n-j}}{\underset{l=(k_{j}-1)2^{n-j}+1}{\sum }}{\dot{x}}_{l}\left(t\right)=p_{\text{in}}\left(t\right),\\ x_{i}\left(0\right)=x_{i0}^{\text{in}},\;0\leq t\leq T_{\text{in}},\;\;i=1,2,\ldots ,2^{n}, \end{array}$$

where *c*_*i*_^in^(*x*_*i*_), *i* = 1,2,…, 2^*n*^, are nonlinear functions of *x*_*i*_, *i* = 1,2,…, 2^*n*^, given by [22]

(4) $$c_{i}^{\text{in}}\left(x_{i}\right)≜\{\begin{array}{cc} a_{i_{1}}^{\text{in}}+b_{i_{1}}^{\text{in}}x_{i}, & \text{if}\;0\leq x_{i}\leq x_{i_{1}}^{\text{in}},\\ a_{i_{2}}^{\text{in}}, & \text{if}\;x_{i_{1}}^{\text{in}}\leq x_{i}\leq x_{i_{2}}^{\text{in}},\\ a_{i_{3}}^{\text{in}}+b_{i_{3}}^{\text{in}}x_{i}, & \text{if}\;x_{i_{2}}^{\text{in}}\leq x_{i}\leq V_{T_{i}}, \end{array}\;i=1,\;\ldots ,\;2^{n},$$

where *a*_*i*_*j*__^in^, *j* = 1,2, 3, and *b*_*i*_*j*__^in^, *j* = 1,3, are model parameters with *b*_*i*_1__^in^ > 0 and *b*_*i*_3__^in^ < 0, *x*_*i*_*j*__^in^, *j* = 1,2, are volume ranges wherein the compliance is constant, *V*_*T*_*i*__ denotes tidal volume, and

(5) $$\begin{array}{c} k_{j}=\left\lfloor \frac{k_{j+1}-1}{2}\right\rfloor \;+1,\;\;j=0,\ldots ,n-1,\;\;\;k_{n}=i, \end{array}$$

where ⌊*q*⌋ denotes the *floor function* which gives the largest integer less than or equal to the positive number *q*.  Figure 3 shows a typical piecewise linear compliance function for inspiration. A similar compliance representation holds for expiration and is also shown in Figure 3.

To further elucidate the inspiration state equation for a 2^*n*^-compartment model, consider the four-compartment model shown in Figure 2 corresponding to a two-generation lung model. Let *x*_*i*_, *i* = 1,2, 3,4, denote the compartmental volumes. Now, the pressure (1/*c*_*i*_^in^(*x*_*i*_  (*t*)))*x*_*i*_(*t*) due to the compliance in *i*th compartment will be equal to the difference between the driving pressure and the resistance to air flow at every airway in the path leading from the pressure source to the *i*th compartment. In particular, for *i* = 3 (see Figure 2),

(6) $$\begin{array}{cc} \frac{1}{c_{3}^{\text{in}}\left(x_{3}\left(t\right)\right)}x_{3}\left(t\right) & =p_{\text{in}}\left(t\right)-R_{0,1}^{\text{in}}\left[{\dot{x}}_{1}\left(t\right)+{\dot{x}}_{2}\left(t\right)+{\dot{x}}_{3}\left(t\right)+{\dot{x}}_{4}\left(t\right)\right]\\ & \;-R_{1,2}^{\text{in}}\left[{\dot{x}}_{3}\left(t\right)+{\dot{x}}_{4}\left(t\right)\right]-R_{2,3}^{\text{in}}{\dot{x}}_{3}\left(t\right), \end{array}$$

or, equivalently,

(7) $$\begin{array}{cc} & R_{2,3}^{\text{in}}{\dot{x}}_{3}\left(t\right)+R_{1,2}^{\text{in}}\left[{\dot{x}}_{3}\left(t\right)+{\dot{x}}_{4}\left(t\right)\right]\\ & \;+R_{0,1}^{\text{in}}\left[{\dot{x}}_{1}\left(t\right)+{\dot{x}}_{2}\left(t\right)+{\dot{x}}_{3}\left(t\right)+{\dot{x}}_{4}\left(t\right)\right]\\ & \;+\frac{1}{c_{3}^{\text{in}}\left(x_{3}\left(t\right)\right)}x_{3}\left(t\right)=p_{\text{in}}\left(t\right). \end{array}$$

Next, we consider the state equation for the expiration process. As in the single-compartment model, we assume that the expiration process is passive and the external pressure applied is *p*_ex_(*t*), *t* ≥ 0. Following an identical procedure as in the inspiration case, we obtain the state equation for expiration as

(8) $$\begin{array}{c} R_{n,i}^{\text{ex}}{\dot{x}}_{i}\left(t\right)+\overset{n-1}{\underset{j=0}{\sum }}R_{j,k_{j}}^{\text{ex}}\overset{k_{j}2^{n-j}}{\underset{l=\left(k_{j}-1\right)2^{n-j}+1}{\sum }}{\dot{x}}_{l}\left(t\right)+\frac{1}{c_{i}^{\text{ex}}\left(x_{i}\left(t\right)\right)}x_{i}\left(t\right)=p_{\text{ex}}\left(t\right),\\ x_{i}\left(T_{\text{in}}\right)=x_{i_{0}}^{\text{ex}},\;T_{\text{in}}\leq t\leq T_{\text{ex}}+T_{\text{in}},\;i=1,2,\ldots ,2^{n}, \end{array}$$

where

(9) ciex(xi)≜{ai1ex+bi1exxi,  ai2ex,ai3ex+bi3exxi,  if  0≤xi≤xi1ex,if  xi1ex≤xi≤xi2ex,if  xi2ex≤xi≤VTi,  i=1,  …,2n,

*a*_*i*_*j*__^ex^, *j* = 1,2, 3, and *b*_*i*_*j*__^ex^, *j* = 1,3, are model parameters with *b*_*i*_1__^ex^ > 0 and *b*_*i*_3__^ex^ < 0, *x*_*i*_*j*__^ex^, *j* = 1,2, are volume ranges wherein the compliance is constant, and *k*_*j*_ is given by (5).

**Figure 4:**
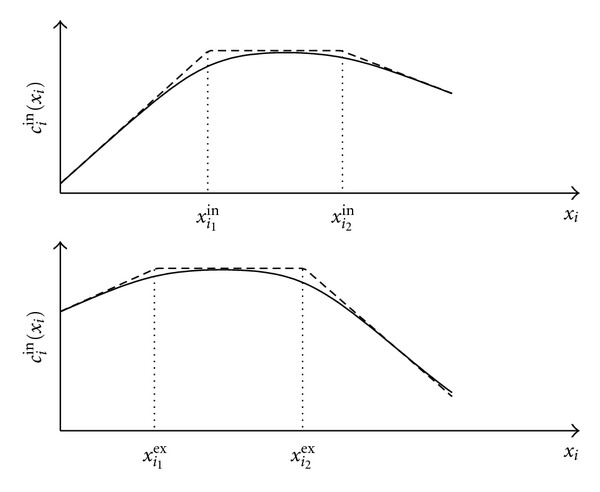
Original and the smoothed compliance functions, *β* = 30.

Next, we provide a smooth (i.e., C^*∞*^) characterization of the nonlinear compliance using sigmoidal functions [23]. Specifically, for inspiration, *c*_*i*_^in^(*x*_*i*_) can be approximated as

(10) $$c_{i}^{\text{in}}\left(x_{i}\right)\approx a_{i_{2}}^{\text{in}}\left(S_{a,b}^{\left(\beta \right)}\left(x_{i}\right)-S_{c,d}^{\left(\beta \right)}\left(x_{i}\right)\right),\;i=1,\ldots ,2^{n},$$

where *a* = −*a*_*i*_1__^in^/*b*_*i*_1__^in^, *b* = (*a*_*i*_2__^in^/*b*_*i*_1__^in^) + *a*, *c* = −*a*_*i*_3__^in^/*b*_*i*_3__^in^, *d* = *a*_*i*_2__^in^/*b*_*i*_3__^in^ + *c*, *S*_*a*,*b*_^(*β*)^(*x*_*i*_)≜1/(*b* − *a*)ln⁡(*σ*_*b*_^(−*β*)^  (*x*_*i*_)/*σ*_*a*_^(−*β*)^  (*x*_*i*_  ))^1/*β*^ with *σ*_*b*_^(−*β*)^(*x*_*i*_)≜1/(1 + *e* ^−*β*(*x*_*i*_−*a*)^), and *β* > 0 is an approximation parameter.  Figure 4 shows the smoothed approximation of the piecewise linear compliance function *c*_*i*_^in^(*x*_*i*_). A similar approximation holds for *c*_*i*_^ex^(*x*_*i*_) and is also shown in Figure 4.

Finally, we rewrite the state equations (3) and (8) for inspiration and expiration, respectively, in vector-matrix state space form. Specifically, define the state vector *x*≜[*x*_1_, *x*_2_,…, *x*_2^*n*^_]^*T*^, where *x*_*i*_ denotes the lung volume of the *i*th compartment. Now, the state equations (3) for inspiration can be rewritten as

(11) $$\begin{array}{c} R_{\text{in}}\dot{x}\left(t\right)+C_{\text{in}}\left(x\left(t\right)\right)x\left(t\right)=p_{\text{in}}\left(t\right)e,\;\;x\left(0\right)=x_{0}^{\text{in}},\;\;0\leq t\leq T_{\text{in}}, \end{array}$$

where **e**≜[1,…,1]^*T*^ denotes the one vector of order 2^*n*^, *C*_in_(*x*) is a diagonal matrix function given by

(12) $$\begin{array}{c} C_{\text{in}}\left(x\right)≜\mathrm{diag}\;\left[\frac{1}{c_{1}^{\text{in}}\left(x_{1}\right)},\ldots ,\frac{1}{c_{2^{n}}^{\text{in}}\left(x_{2^{n}}\right)}\right] \end{array},$$

(13) $$\begin{array}{c} R_{\text{in}}≜\overset{n}{\underset{j=0}{\sum }}\;\overset{2^{j}}{\underset{k=1}{\sum }}R_{j,k}^{\text{in}}Z_{j,k}Z_{j,k}^{\text{T}}, \end{array}$$

where *Z*_*j*,*k*_ ∈ ℝ^2^*n*^^ is such that the *l*th element of *Z*_*j*,*k*_ is 1 for all *l* = (*k* − 1)2^*n*−*j*^ + 1, (*k* − 1)2^*n*−*j*^ + 2,…, *k*2^*n*−*j*^, *k* = 1,…, 2^*j*^, *j* = 0,1,…, *n*, and zero elsewhere.

Similarly, the state equation (8) for expiration can be rewritten as

(14) $$\begin{array}{c} R_{\text{ex}}\dot{x}\left(t\right)+C_{\text{ex}}\left(x\left(t\right)\right)x\left(t\right)=p_{\text{ex}}\left(t\right)e,\;\;\;x\left(T_{\text{in}}\right)=x_{0}^{\text{ex}},\\ T_{\text{in}}\leq t\leq T_{\text{ex}}+T_{\text{in}}, \end{array}$$

where

(15) $$\begin{array}{c} C_{\text{ex}}\left(x\right)≜\mathrm{diag}\left[\frac{1}{c_{1}^{\text{ex}}\left(x_{1}\right)},\ldots ,\frac{1}{c_{2^{n}}^{\text{ex}}\left(x_{2^{n}}\right)}\right], \end{array}$$

(16) $$\begin{array}{c} R_{\text{ex}}≜\overset{n}{\underset{j=0}{\sum }}\;\overset{2^{j}}{\underset{k=1}{\sum }}R_{j,k}^{\text{ex}}\;Z_{j,k}Z_{j,k}^{\text{T}}. \end{array}$$

Finally, it follows from [6, Proposition 4.1] that *R*_in_ and *R*_ex_ are positive definite and, hence, *R*_in_ and *R*_ex_ are invertible matrices.

## 3. Optimal Determination of Inspiratory and Expiratory Airflow in Breathing

 In this section, we use the respiratory dynamical system characterized by (11) and (14) to develop an optimal model for predicting airflow patterns in breathing. The optimization criteria used allows for the minimization of oxygen expenditure of the respiratory muscles as well as rapid changes in the lung volume flow rate. The oxygen consumption of the lung muscles is mainly due to the work carried out by the respiratory muscles to overcome the resistive forces and stretch the lung and chest wall. In [24], this work is defined as *PV*, where *P* is the pressure driving inflation and *V* is the lung unit volume. The efficiency of gas exchange in the lungs is related to the volume acceleration, since rapid changes in lung volume can cause discomfort and inefficacy of muscular contraction and control. Moreover, high-volume acceleration can result in overexpansion of the lung resulting in lung tissue rupture as well as excessive work of breathing with subsequent ventilatory muscle fatigue.

In the ensuing discussion, we assume that the inspiration process starts from a given initial state *x*_0_^in^ and is followed by the expiration process where its initial state will be the final state of the inspiration. An inspiration followed by an expiration is called a single *breathing cycle*. Furthermore, we assume that each breathing cycle is followed by another breathing cycle where the initial condition for the latter breathing cycle is the final state of the former breathing cycle. Since the respiratory process is periodic, we need only focus on one breathing cycle.

The next result gives the optimal solution x*(*t*), 0 ≤ *t* ≤ *T*_in_, for the inspiratory airflow breathing pattern using classical calculus of variations techniques.

**Theorem 1**

Consider the nonlinear system model for inspiration given by (11). Let the optimal air volume x*(*t*), 0 ≤ *t* ≤ *T*_in_, be given by the solution to the minimization problem

(17) $$\begin{array}{c} {𝒥}_{\text{in}}\left(x\right)=\overset{T_{\text{in}}}{\underset{0}{\int }}\left[{\ddot{x}}^{T}\left(t\right)\ddot{x}\left(t\right)+\alpha_{1}p_{\text{in}}\left(t\right)e^{T}\dot{x}\left(t\right)\right]\text{d}t,\;\alpha_{1}\geq 0, \end{array}$$

subject to the natural boundary conditions

(18) $$x\left(0\right)=V_{0},\;\dot{x}\left(0\right)=0,$$

(19) $$x\left(T_{\text{in}}\right)=V_{0}+V_{T},\;\;\dot{x}\left(T_{\text{in}}\right)=0,$$

where *V*_0_ ∈ ℝ^2^*n*^^ is the end expiratory volume and *V*_*T*_ ∈ ℝ^2^*n*^^ is the tidal volume. If *α*_1_ > 0, then x*(*t*),  0 ≤ *t* ≤ *T*_in_, is given by

(20) $$\begin{array}{cc} x^{*}\left(t\right) & =d_{1}+d_{2}t+\exp\left(\sqrt{\alpha_{1}}R_{\text{in}}^{1/2}t\right)d_{3}\\ & \;+\exp\left(-\sqrt{\alpha_{1}}R_{\text{in}}^{1/2}t\right)d_{4},\;t\geq 0, \end{array}$$

and if *α*_1_ = 0, then

(21) $$\begin{array}{c} x^{*}\left(t\right)=d_{1}+d_{2}t+d_{3}t^{2}+d_{4}t^{3},\;\;t\geq 0, \end{array}$$

where *d*_1_, *d*_2_, *d*_3_, and *d*_4_ ∈ ℝ^2^*n*^^ are constant vectors determined by the boundary conditions (18) and (19), and *R*_in_^1/2^ denotes the (unique) positive-definite square root of *R*_in_.

**Proof**

First, note that *p*_in_(*t*)**e**, 0 ≤ *t* ≤ *T*_in_, in (17) can be eliminated using the state equation (11). Thus, the integrand of the performance criterion (17) can be written as

(22) $$\begin{array}{cc} & L_{\text{in}}\left(x\left(t\right),\dot{x}\left(t\right),\ddot{x}\left(t\right)\right)\\ & \;\;={\ddot{x}}^{T}\left(t\right)\ddot{x}\left(t\right)\\ & \;\;\;+\alpha_{1}{\left[R_{\text{in}}\dot{x}\left(t\right)+C_{\text{in}}\left(x\left(t\right)\right)x\left(t\right)\right]}^{T}\dot{x}\left(t\right)\\ & \;\;={\ddot{x}}^{T}\left(t\right)\ddot{x}\left(t\right)\\ & \;\;\;+\alpha_{1}\left[{\dot{x}}^{T}\left(t\right)R_{\text{in}}\dot{x}\left(t\right)+x^{T}\left(t\right)C_{\text{in}}\left(x\right)\dot{x}\left(t\right)\right],\\ & {\;\;\;\;\;\;\;\;\;\;\alpha }_{1}\geq 0. \end{array}$$

The first variation of the performance criterion *𝒥*_in_(*x*) is given by

(23) $$\begin{array}{cc} \delta {𝒥}_{\text{in}}\left(x^{*},\delta x\right) & =\overset{T_{\text{in}}}{\underset{0}{\int }}\delta L_{\text{in}}\left(x^{*}\left(t\right),{\dot{x}}^{*}\left(t\right),{\ddot{x}}^{*}\left(t\right)\right)\text{d}t\\ & =\overset{T_{\text{in}}}{\underset{0}{\int }}\{\left(\frac{\partial L_{\text{in}}}{\partial x}\right)\delta x\left(t\right)+\left(\frac{\partial L_{\text{in}}}{\partial \dot{x}}\right)\delta \dot{x}\left(t\right)\\ & \;\;\;\;\;+\left(\frac{\partial L_{\text{in}}}{\partial \ddot{x}}\right)\delta \ddot{x}\left(t\right)\}\text{d}t\\ & ={\left[\frac{\partial L_{\text{in}}}{\partial \ddot{x}}\delta \dot{x}+\left(\frac{\partial L_{\text{in}}}{\partial \dot{x}}-\frac{{\text{d}}^{2}}{\text{d}t^{2}}\frac{\partial L_{\text{in}}}{\partial \ddot{x}}\right)\delta x\right]}_{0}^{T_{\text{in}}}\\ & \;+\overset{T_{\text{in}}}{\underset{0}{\int }}\{\left(\frac{\partial L_{\text{in}}}{\partial x}\right)-\frac{\text{d}}{\text{d}t}\left(\frac{\partial L_{\text{in}}}{\partial \dot{x}}\right)\\ & \;\;\;\;\;+\frac{\text{d}}{\text{d}t}\left(\frac{\partial L_{\text{in}}}{\partial \ddot{x}}\right)\}\delta x\left(t\right)\text{d}t. \end{array}$$

Using the boundary conditions (18) and (19), it follows that $\delta x(0)=\delta x(T_{\text{in}})=\delta \dot{x}(0)=\delta \dot{x}(T_{\text{in}})=0$. Now, since *T*_in_ is fixed, it follows from the fundamental theorem of the calculus of variations that the variation of *𝒥*_in_(*x*) must vanish on x*; that is, the extremals optimizing the performance criterion *𝒥*_in_(*x*) satisfy the Euler-Lagrange equation

(24) $$\begin{array}{c} {\left(\frac{\partial L_{\text{in}}}{\partial x}\right)}^{T}-\frac{\text{d}}{\text{d}t}{\left(\frac{\partial L_{\text{in}}}{\partial \dot{x}}\right)}^{T}+\frac{{\text{d}}^{2}}{\text{d}t^{2}}{\left(\frac{\partial L_{\text{in}}}{\partial \ddot{x}}\right)}^{T}=0. \end{array}$$

Next, using *C*_in_(*x*) given by (12),

(25) $$\begin{array}{cc} {\left(\frac{\partial L_{\text{in}}}{\partial x}\right)}^{T} & =\alpha_{1}C_{\text{in}}\left(x\left(t\right)\right)\dot{x}\left(t\right)\\ & \;+\alpha_{1}C_{\text{in}}^{\prime }\left(x\left(t\right)\right)\dot{X}\left(t\right)x\left(t\right),\;0\leq t\leq T_{\text{in}},\\ {\left(\frac{\partial L_{\text{in}}}{\partial \dot{x}}\right)}^{T} & =2\alpha_{1}R_{\text{in}}\dot{x}\left(t\right)+\alpha_{1}C_{\text{in}}\left(x\left(t\right)\right)x\left(t\right),\;\;0\leq t\leq T_{\text{in}},\\ {\left(\frac{\partial L_{\text{in}}}{\partial \ddot{x}}\right)}^{T} & =2\ddot{x}\left(t\right),\;\;0\leq t\leq T_{\text{in}}, \end{array}$$

where *C*_in_′(*x*(*t*))≜diag⁡  [(∂/∂*x*_*i*_)(1/(*c*_*i*_^in^  (*x*_*i*_  (*t*))))] and $\dot{X}\left(t\right)≜\mathrm{diag}\left[{\dot{x}}_{i}(t)\right],i=1,\ldots ,2^{n}$. Thus, (24) yields the fourth-order differential equation

(26) $$\begin{array}{c} x^{\left(4\right)}\left(t\right)-\alpha_{1}R_{\text{in}}x^{\left(2\right)}\left(t\right)=0,\;\;0\leq t\leq T_{\text{in}}, \end{array}$$

where *x* ^(*n*)^(*t*)≜(d^*n*^ *x*(*t*)/d*t* ^*n*^  ), with boundary conditions given in (18) and (19). Now, using standard analysis techniques, the solution *x*(*t*),  0 ≤ *t* ≤ *T*_in_, to (26) satisfies (20) if *α*_1_ > 0 and (21) if *α*_1_ = 0.

**Remark 2**

The vectors *d*_1_, *d*_2_, *d*_3_, and d_4_ in Theorem 1 can be uniquely determined using the four boundary conditions given by (18) and (19). Specifically, if *α*_1_ = 0, it can be shown that *d*_1_ = *V*_0_, *d*_2_ = 0, *d*_3_ = (3/*T*_in_^2^  )*V*_*T*_, and *d*_4_ = −(2/*T*_in_^3^  )*V*_*T*_. Hence, in this case, $\dot{x}\left(t\right)$ = *d*_2_ + 2*d*_3_ *t* + 3*d*_4_ *t* ^2^ = (6*t*/(*T*_in_^2^  ))*V*_*T*_(1 − (*t*/*T*_in  _))≥≥0, 0 ≤ *t* ≤ *T*_in_, which implies that the solution x*(*t*), 0 ≤ *t* ≤ *T*_in_, to (26) is increasing during inspiration, and hence, *V*_0_*i*__ ≤ *x*_i_*(*t*) ≤ *V*_0_*i*__ + *V*_*T*_*i*__, *i* = 1,…, 2^*n*^, where *V*_0_*i*__,  *x*_*i*_(*t*) and *V*_*T*_*i*__ are the *i*th components of *V*_0_, *x*(*t*), and *V*_*T*_, respectively. A similar result holds for the case where *α*_1_ > 0.

Next, we give the optimal solution x*(*t*), *T*_in_ ≤ *t* ≤ *T*_in_ + *T*_ex_, for the expiratory airflow breathing pattern.

**Theorem 3**

Consider the nonlinear system model for expiration given by (14). Let the optimal solution x*(*t*), *T*_in_ ≤ *t* ≤ *T*_in_ + *T*_ex_, be given by the solution to the minimization problem

(27) $$\begin{array}{cc} {𝒥}_{\text{ex}}\left(x\right) & =\overset{T_{\text{in}}+T_{\text{ex}}}{\underset{T_{\text{in}}}{\int }}\left[{\ddot{x}}^{T}\left(t\right)\ddot{x}\left(t\right)+\alpha_{2}p_{\text{ex}}^{2}\left(t\right)e^{T}e\right]\text{d}t,\;\;\alpha_{2}\geq 0, \end{array}$$

subject to the natural boundary conditions

(28) $$x\left(T_{\text{in}}\right)=V_{0}+V_{T},\;\;\dot{x}\left(T_{\text{in}}\right)=0,$$

(29) $$x\left(T_{\text{in}}+T_{\text{ex}}\right)=V_{0},\;\;\dot{x}\left(T_{\text{in}}+T_{\text{ex}}\right)=0.$$

If *α*_2_ > 0, then x*(*t*), *T*_in_ ≤ *t* ≤ *T*_in_ + *T*_ex_, satisfies

(30) $$\begin{array}{cc} & x^{\left(4\right)}\left(t\right)-\alpha_{2}R_{\text{ex}}^{2}x^{\left(2\right)}\left(t\right)+\alpha_{2}C_{\text{ex}}^{2}\left(x\right)x\left(t\right)\\ & \;+\alpha_{2}[C_{\text{ex}}\left(x\right)R_{\text{ex}}\dot{x}\left(t\right)-R_{\text{ex}}C_{\text{ex}}\left(x\right)\dot{x}\left(t\right)\\ & \;\;\;\;+X\left(t\right)C_{\text{ex}}^{\prime }\left(x\right)R_{\text{ex}}\dot{x}\left(t\right)\\ & \;\;\;\;-R_{\text{ex}}C_{\text{ex}}^{\prime }\left(x\right)X\left(t\right)\dot{x}\left(t\right)\\ & \;\;\;\;+X\left(t\right)C_{\text{ex}}^{\prime }\left(x\right)C_{\text{ex}}\left(x\right)x\left(t\right)]=0, \end{array}$$

where *X*(*t*)≜diag⁡[*x*_*i*_(*t*)] and *C*_ex_′(*x*)≜diag⁡[(∂/∂*x*_*i*_)(1/*c*_*i*_^ex  ^(*x*_*i*_))],   *i* = 1,…, 2^*n*^, and if *α*_2_ = 0, then

(31) $$\begin{array}{c} x^{*}\left(t\right)=d_{1}+d_{2}t+d_{3}t^{2}+d_{4}t^{3},\;\;t\geq 0, \end{array}$$

where *d*_1_, *d*_2_, *d*_3_, and *d*_4_ ∈ ℝ^2^*n*^^ are constant vectors determined by the four boundary conditions (28) and (29).

**Proof**

Using (14), the integrand of the performance criterion (27) can be written as

(32) $$\begin{array}{cc} L_{\text{ex}}\left(x\left(t\right),\dot{x}\left(t\right),\ddot{x}\left(t\right)\right) & ={\ddot{x}}^{T}\left(t\right)\ddot{x}\left(t\right)+\alpha_{2}{\left(p_{\text{ex}}\left(t\right)e\right)}^{T}\left(p_{\text{ex}}\left(t\right)e\right)\\ & ={\ddot{x}}^{T}\left(t\right)\ddot{x}\left(t\right)\\ & \;+\alpha_{2}{\left[R_{\text{ex}}\dot{x}\left(t\right)+C_{\text{ex}}\left(x\left(t\right)\right)x\left(t\right)\right]}^{T}\\ & \;\times \left[R_{\text{ex}}\dot{x}\left(t\right)+C_{\text{ex}}\left(x\left(t\right)\right)x\left(t\right)\right]\\ & ={\ddot{x}}^{T}\left(t\right)\ddot{x}\left(t\right)\\ & \;+\alpha_{2}[{\dot{x}}^{T}\left(t\right)R_{\text{ex}}^{2}\dot{x}\left(t\right)+x^{T}\left(t\right)\\ & \;\;\;\;\times C_{\text{ex}}^{2}\left(x\left(t\right)\right)x\left(t\right)\\ & \;\;\;\;+2{\dot{x}}^{T}\left(t\right)R_{\text{ex}}C_{\text{ex}}\left(x\left(t\right)\right)x\left(t\right)],\\ & \;\;\;\;\;\;\;\;\;\;\alpha_{2}>0. \end{array}$$

Thus, the variation of *𝒥*_ex_(*x*) on an extremal solution gives

(33) $$\begin{array}{cc} \delta {𝒥}_{\text{ex}}\left(x^{*},\delta x\right) & =\overset{T_{\text{in}}+T_{\text{ex}}}{\underset{T_{\text{in}}}{\int }}\delta L_{\text{ex}}\left(x^{*}\left(t\right),{\dot{x}}^{*}\left(t\right),{\ddot{x}}^{*}\left(t\right)\right)\text{d}t\\ & =\overset{T_{\text{in}}+T_{\text{ex}}}{\underset{T_{\text{in}}}{\int }}\{\left(\frac{\partial L_{\text{ex}}}{\partial x}\right)\delta x\left(t\right)+\left(\frac{\partial L_{\text{ex}}}{\partial \dot{x}}\right)\delta \dot{x}\left(t\right)\\ & \;\;\;\;\;+\left(\frac{\partial L_{\text{ex}}}{\partial \ddot{x}}\right)\delta \ddot{x}\left(t\right)\}\text{d}t\\ & ={\left[\frac{\partial L_{\text{ex}}}{\partial \ddot{x}}\delta \dot{x}+\left(\frac{\partial L_{\text{ex}}}{\partial \dot{x}}-\frac{\text{d}}{\text{d}t}\frac{\partial L_{\text{ex}}}{\partial \ddot{x}}\right)\delta x\right]}_{T_{\text{in}}}^{T_{\text{in}}+T_{\text{ex}}}\\ & \;+\overset{T_{\text{ex}}}{\underset{0}{\int }}\{\left(\frac{\partial L_{\text{ex}}}{\partial x}\right)-\frac{\text{d}}{\text{d}t}\left(\frac{\partial L_{\text{ex}}}{\partial \dot{x}}\right)\\ & \;\;\;\;+\frac{{\text{d}}^{2}}{\text{d}t^{2}}\left(\frac{\partial L_{\text{ex}}}{\partial \ddot{x}}\right)\}\delta x\left(t\right)\text{d}t=0. \end{array}$$

Using the boundary conditions (28) and (29), it follows that $\delta x(T_{\text{in}})=\delta x(T_{\text{in}}+T_{\text{ex}})=\delta \dot{x}(T_{\text{in}})=\delta \dot{x}(T_{\text{in}}+T_{\text{ex}})=0$. Hence, the extremals optimizing the performance criterion *𝒥*_ex_(*x*) satisfy the Euler-Lagrange equation

(34) $$\begin{array}{c} {\left(\frac{\partial L_{\text{ex}}}{\partial x}\right)}^{T}-\frac{\text{d}}{\text{d}t}{\left(\frac{\partial L_{\text{ex}}}{\partial \dot{x}}\right)}^{T}+\frac{{\text{d}}^{2}}{\text{d}t^{2}}{\left(\frac{\partial L_{\text{ex}}}{\partial \ddot{x}}\right)}^{T}=0. \end{array}$$

Now, using *C*_ex_(*x*) given by (15),

(35) $$\begin{array}{cc} {\left(\frac{\partial L_{\text{ex}}}{\partial x}\right)}^{T} & =\alpha_{2}[2C_{\text{ex}}^{2}\left(x\left(t\right)\right)x\left(t\right)+2C_{\text{ex}}\left(x\left(t\right)\right)R_{\text{ex}}\dot{x}\left(t\right)\\ & \;\;\;+2X\left(t\right)C_{\text{ex}}^{\prime }\left(x\left(t\right)\right)R_{\text{ex}}\dot{x}\left(t\right)\\ & \;\;\;+2X\left(t\right)C_{\text{ex}}^{\prime }\left(x\left(t\right)\right)C_{\text{ex}}\left(x\left(t\right)\right)x\left(t\right)],\;\\ & \;\;\;\;\;\;\;\;T_{\text{in}}\leq t\leq T_{\text{in}}+T_{\text{ex}},\\ {\left(\frac{\partial L_{\text{ex}}}{\partial \dot{x}}\right)}^{T} & =\alpha_{2}\left[2R_{\text{ex}}^{2}\dot{x}\left(t\right)+2R_{\text{ex}}C_{\text{ex}}\left(x\left(t\right)\right)x\left(t\right)\right],\\ & \;\;\;\;\;\;\;\;T_{\text{in}}\leq t\leq T_{\text{in}}+T_{\text{ex}},\\ {\left(\frac{\partial L_{\text{ex}}}{\partial \ddot{x}}\right)}^{T} & =2\ddot{x}\left(t\right),\;\;T_{\text{in}}\leq t\leq T_{\text{in}}+T_{\text{ex}}, \end{array}$$

which yields (30). Finally, in the case where *α*_2_ = 0, (30) collapses to *x* ^(4)^(*t*) = 0, *T*_in_ ≤ *t* ≤ *T*_in_ + *T*_ex_, which satisfies (31).

**Remark 4**

In the case where *α*_2_ = 0, the vectors *d*_1_, *d*_2_, *d*_3_, and *d*_4_ in Theorem 3 can be uniquely determined using the four boundary conditions (28) and (29). In particular, *d*_1_ = *V*_0_ + *V*_*T*_ + 3*βT*_in_^2^ *T*_ex_ *V*_*T*_ + 2*βT*_in_^3^ *V*_*T*_, *d*_2_ = −*β*(6*T*_in_^2^ *V*_*T*_ + 6*T*_ex_ *T*_in_ *V*_*T*_), *d*_3_ = *β*(3*T*_ex_ *V*_*T*_ + 6*T*_in_ *V*_*T*_), and *d*_4_ = −2*βV*_*T*_, where *β* = 1/(3*T*_ex_^3^ + 12*T*_ex_^2^ *T*_in_ + 12*T*_ex_ *T*_in_^2^ + 4*T*_in_^3^). Hence, $\dot{x}(t)=d_{2}+2d_{3}t+3d_{4}t^{2}=-6\beta V_{T}t(T_{\text{in}}+T_{\text{ex}}-t)-6\beta V_{T}t(t-T_{\text{in}})\leq \leq 0$, *T*_in_ ≤ *t* ≤ *T*_in_ + *T*_ex_, which implies that the solution x*(*t*), *T*_in_ ≤ *t* ≤ *T*_in_ + *T*_ex_, is decreasing during expiration, and hence, *V*_0_*i*__ ≤ *x*_i_*(*t*) ≤ *V*_0_*i*__ + *V*_*T*_*i*__, *i* = 1,…, 2^*n*^. The case where *α*_2_ > 0 involves the solution to (30), and hence, we have been unable to show that x*(*t*), *T*_in_ ≤ *t* ≤ *T*_in_ + *T*_ex_, is decreasing during expiration analytically. However, this has been verified numerically.

**Remark 5**

If optimal solutions to Theorems 1 and 3 exist, then the optimal respiratory airflow patterns and their corresponding driving pressures can be computed using the lung mechanics model developed in Section 2. The input signal to this model can then be used as the driving pressure of a mechanical ventilator needed to achieve the optimal respiratory airflow pattern.

The physiological interpretations of the performance criteria for inspiration and expiration used in Theorems 1 and 3 are slightly different. In particular, the inspiratory criterion *𝒥*_in_(*x*) involves a weighted sum of squares of the lung volume acceleration and the mechanical work performed by the inspiratory muscles. Alternatively, during the expiratory phase, the respiratory muscles remain active in the beginning of expiration since they continue their action by opposing expiration and hence consume oxygen thereby performing negative work. Thus, mechanical work alone is not a satisfactory criterion for describing control of breathing at rest. As in [25], we assume that oxygen consumption of expiration correlates with the integral square of the driving pressure. This assumption is supported in [26] which shows that an index of average respiratory pressure can predict the total oxygen cost of breathing. Hence, instead of mechanical work, we use the integral square of the applied pressure in the expiratory criterion *𝒥*_ex_(*x*), which corresponds to minimizing the mean standard potential energy in the lung.

It can be seen that the optimal solutions x*(*t*), *t* ≥ 0, depend on the variables *T*_in_, *T*_ex_, *V*_0_, and *V*_*T*_ through the boundary conditions. Moreover, the nonlinearities in (30) are due to nonlinearities in the lung compliance *C*_ex_(*x*), which make analytical solutions to (30) difficult to obtain. It is interesting to note that although the optimal solutions x*(*t*), *T*_in_ ≤ *t* ≤ *T*_in_ + *T*_ex_, to (30) during the expiration phase depend on the nonlinear compliance of *C*_ex_(*x*), the optimal solutions x*(*t*), 0 ≤ *t* ≤ *T*_in_, to (26) during the inspiration phase are independent of the nonlinear system compliance *C*_in_(*x*). In the case where *n* = 0 (i.e., a single-lung-compartment model), *x*(*t*) ∈ ℝ, *R*_ex_ ∈ ℝ, and *C*_ex_(*x*) = *C*_ex_ are constants, (30) reduces to

(36) $$\begin{array}{c} x^{\left(4\right)}\left(t\right)-\alpha_{2}R_{\text{ex}}^{2}x^{\left(2\right)}\left(t\right)+\alpha_{2}C_{\text{ex}}^{2}x\left(t\right)=0. \end{array}$$

This case is extensively discussed in [25] wherein the authors characterize four different solutions to (36) corresponding to *α*_2_ = 0, 0 < *α*_2_ < 4*C*_ex_^2^/*R*_ex_^4^, *α*_2_ = 4*C*_ex_^2^/*R*_ex_^4^, and *α*_2_ > 4*C*_ex_^2^/*R*_ex_^4^.

## 4. Numerical Determination of Optimal Volume Trajectories

 The optimal volume trajectories formulated in Section 3 result in two-point nonlinear boundary-value problems. Numerical methods for solving such problems include shooting methods [27] and steepest descent methods [28]. In this section, we use the collocation method implemented by *bvp4c* in MATLAB^  ^ [29] to numerically integrate the differential equations (26) and (30) to obtain the optimal volume trajectory x*(*t*), *t* ≥ 0.

For our simulations, we first consider a two-compartment lung model and use the values for the lung compliance found in [22]. In particular, we set *a*_*i*_1__^in^ = 0.018 *ℓ*/cm H_2_O, *b*_*i*_1__^in^ = 0.0233, *a*_*i*_2__^in^ = 0.025 *ℓ*/cm H_2_O, *a*_*i*_3__^in^ = 0.2532 *ℓ*/cm H_2_O, *b*_*i*_3__^in^ = −0.01, *x*_*i*_1__^in^ = 0.3 *ℓ*, *x*_*i*_2__^in^ = 0.48 *ℓ*, *a*_*i*_1__^ex^ = 0.02 *ℓ*/cm H_2_O, *b*_*i*_1__^ex^ = 0.078, *a*_*i*_2__^ex^ = 0.038 *ℓ*/cm H_2_O, *a*_*i*_3__^ex^ = 0.1025 *ℓ*/cm H_2_O, *b*_*i*_3__^ex^ = −0.15, *x*_*i*_1__^ex^ = 0.23 *ℓ*, *x*_*i*_2__^ex^ = 0.43 *ℓ*, and *i* = 1,2. Here, we assume that the bronchial tree has a dichotomy structure (see Section 2). The airway resistance varies with the branch generation, and typical values can be found in [30]. Furthermore, the expiratory resistance will be higher than the inspiratory resistance by a factor 2 to 3. Here, we assume that the factor is 2.5.

**Figure 5:**
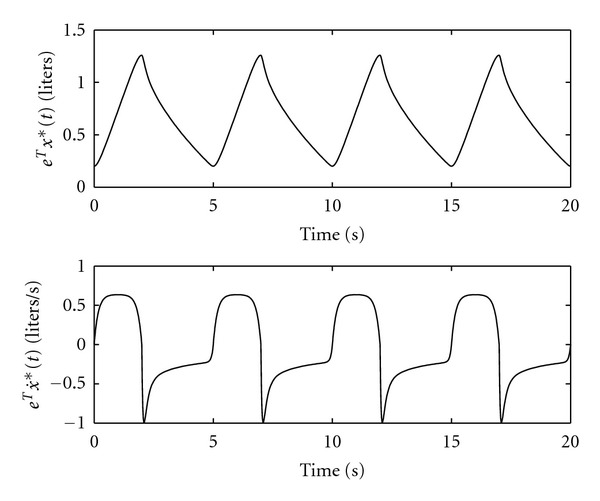
Volume and airflow rate patterns for the total lung compartments.

For our simulation, we assume that the inspiration time *T*_in_ = 2 sec and the expiration time *T*_ex_ = 3 sec. The two weighting parameters *α*_1_ and *α*_2_ differ from person to person. Nominal values for the weighting parameters are *α*_1_ = 2.0*l*/sec^3^⁡ cm H_2_O and *α*_2_ = 0.1 *l* ^2^/sec^4^⁡ cm H_2_O, which correspond to spontaneous breathing at rest [25].  Figure 5 shows the optimal air volume **e** ^*T*^ x*(*t*), *t* ≥ 0, and the optimal airflow rate $e^{T}{\dot{x}}^{*}(t),\;\;t\geq 0$, given by the two-point nonlinear boundary-value problems (24) and (34). Note that the airflow curve for inspiration is symmetric, since the nonlinearities in *C*_in_(*x*) do not appear in (26). However, x*(*t*), *t* ≥ 0, obtained using (30) during expiration involves *C*_ex_(*x*), and hence, the airflow curve is asymmetric.

**Figure 6:**
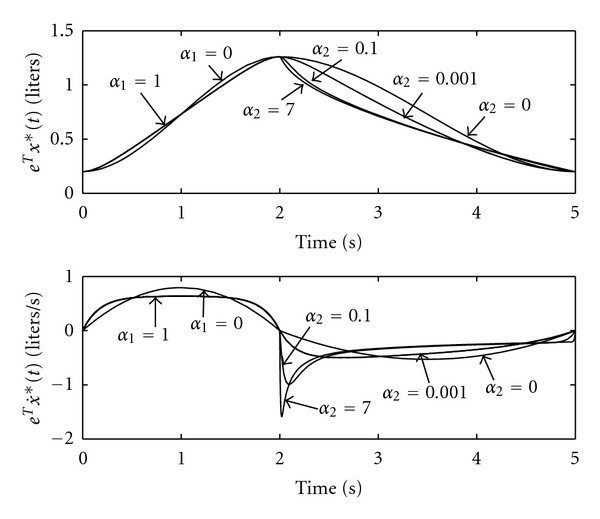
Volume and airflow rate patterns for different *α*_1_'s and *α*_2_'s.

Figure 6 shows the sensitivity of the optimal volume and airflow rate patterns to changes in the parameters *α*_1_ and *α*_2_. As can be seen from the figure, the inspiratory airflow rate is symmetric and the maximum value of the airflow rate decreases as a function of increasing *α*_1_. Furthermore, the asymmetric pattern of the expiratory airflow rate reflects the fact that the minimum value becomes steeper with increasing *α*_2_. Specifically, if we set the weighting parameter *α*_2_ = 0, it follows from (30) that the airflow curve for the expiration is given by a parabolic arc. The airflow patterns in Figure 6 exhibit typical respiratory characteristics observed in spontaneous breathing, that is, the inspiratory airflow rate curve is relatively flat and the expiratory airflow rate waveform is asymmetric with an initial trough, and quite similar to “real” airflow signals [31].

**Figure 7:**
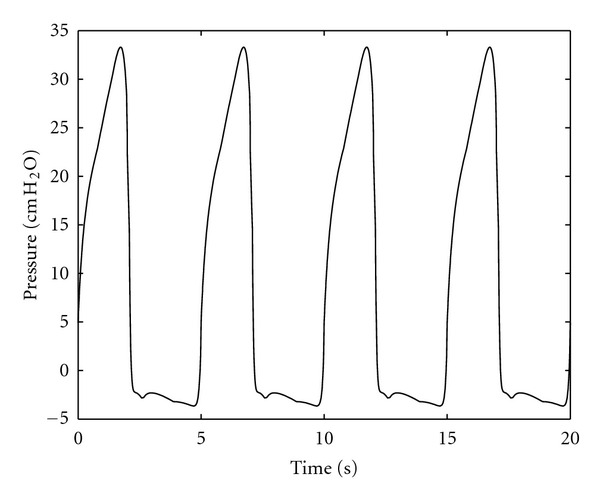
Pressure generated by optimal solution.

**Figure 8:**
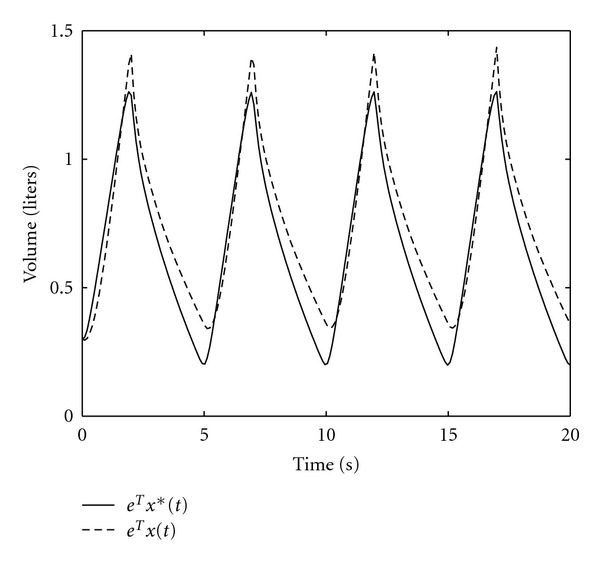
Optimal volume **e** ^*T*^ x*(*t*) and nonoptimal volume **e** ^*T*^ *x*(*t*) versus time.

**Figure 9:**
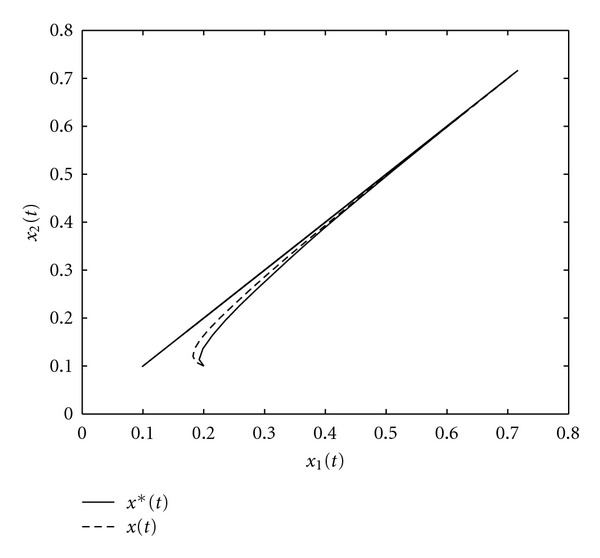
Phase portrait for *x*_1_*(*t*) versus *x*_2_*(*t*) and *x*_1_(*t*) versus *x*_2_(*t*).

**Figure 10:**
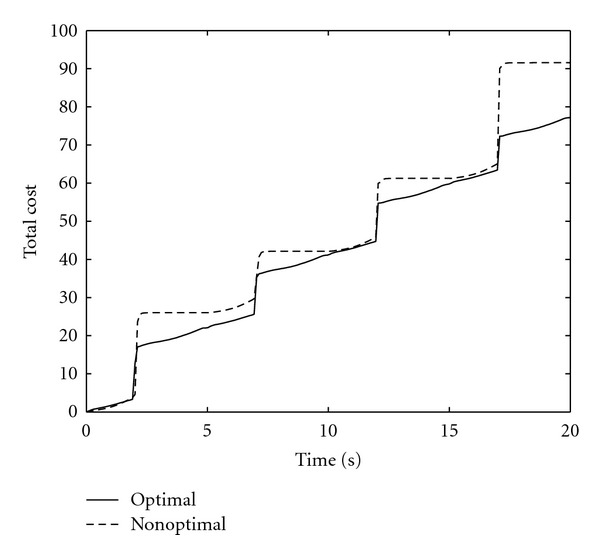
Performance criterion comparison versus time.

Figure 7 shows the driving pressure generated by the respiratory muscles using the optimal air volume **e** ^*T*^ x*(*t*), *t* ≥ 0.  Figure 8 compares the optimal air volume trajectory **e** ^*T*^ x*(*t*), *t* ≥ 0, with a nonoptimal air volume trajectory **e** ^*T*^ *x*(*t*), *t* ≥ 0, generated by the linear pressure *p*_in_(*t*) = 20*t* + 5 cm H_2_O, *t* ∈ [0, *T*_in_], and *p*_ex_(*t*) = 0 cm H_2_O, *t* ∈ [*T*_in_, *T*_in_ + *T*_ex_], [6]. Note that **e** ^*T*^ x*(*t*), *t* ≥ 0, switches between the end expiratory level **e** ^*T*^ *V*_0_ = 0.2 *l* and the tidal volume **e** ^*T*^ *V*_*T*_ = 1.2 *l*.  Figure 9 shows the phase portrait of the optimal trajectories *x*_1_*(*t*) and *x*_2_*(*t*) and suboptimal trajectories *x*_1_(*t*) and *x*_2_(*t*). Note that both sets of trajectories asymptotically converge to a limit cycle, with the optimal solutions satisfying the boundary conditions given in (18), (19), (28), and (29).  Figure 10 compares the value of the total performance criterion generated by the optimal air volume with the value of the total performance criterion generated by the nonoptimal air volume.

**Figure 11:**
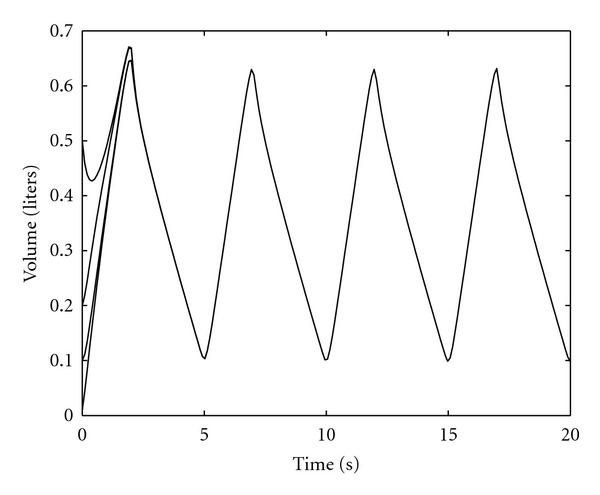
Optimal volume x*(*t*) versus time for a four-compartmental model.

Finally, Figure 11 shows the optimal air volume trajectories for a four-compartment model with each air volume trajectory satisfying the boundary conditions given in (18), (19), (28), and (29). For this simulation, the compliance parameters are taken to be identical to those used for the two-compartment model with *i* = 1,2, 3,4, and the values for airway resistances are generated using the results of [30].

## 5. Conclusion and Directions for Future Work

 In this paper, we developed an optimal respiratory air flow pattern using a nonlinear multicompartment model for a lung mechanics system. The determination of the optimal air volume trajectories is derived using classical calculus of variations techniques and involves optimization criteria that account for oxygen expenditure of the respiratory lung muscles, lung volume acceleration, and elastic potential energy of the lung. Future work will include the development of multivariable and adaptive control algorithms that will utilize these models within a model reference control architecture for fully automating mechanical ventilation to ensure adequate ventilation and oxygenation for critical care patients in intensive care units.

Since sedation in intensive care units is often administered to prevent the patient from fighting the ventilator, it seems plausible to use respiratory parameters as a performance variable for closed-loop control. Calculation of patient work of breathing requires measurement of a patient-generated pressure/volume loop or work of breathing. Since work of breathing can be measured using a commercially available esophageal balloon [32], work of breathing can serve as a performance variable for closed-loop control of sedation. Furthermore, patient-ventilator dyssynchrony can be identified by analysis of pressure/flow wave forms [33].

Closed-loop control algorithms can use either work of breathing as measured by an esophageal balloon or patient respiratory rate as a performance variable for closed-loop control of sedation. The need for optimal control algorithms is necessary for achieving a target performance value while satisfying certain constraints. For example, we could seek to design a control algorithm that seeks to minimize the patient respiratory rate (above the set ventilator rate) but which does not result in hypotension. This requires the development of a constrained optimal control framework that seeks to minimize a given performance measure (e.g., patient respiratory rate) within a class of fixed-architecture controllers satisfying internal controller constraints (e.g., controller order, control signal nonnegativity, etc.) as well as system constraints (e.g., blood pressure, system state nonnegativity, etc.). The results in the present paper can serve as a starting point for developing multivariable controllers for mechanical ventilation of critically ill patients.
